# Embedding the Future of Regenerative Medicine into the Open Epigenomic Landscape of Pluripotent Human Embryonic Stem Cells

**Published:** 2013

**Authors:** Xuejun H. Parsons

**Affiliations:** 1 San Diego Regenerative Medicine Institute, San Diego, CA 92109, USA; 2 *Xcelthera, San Diego, CA 92109, USA*

**Keywords:** Human embryonic stem cell, stem cell, pluripotent, epigenome, chromatin, regenerative medicine, neurological disease, heart disease, cell therapy

## Abstract

It has been recognized that pluripotent human embryonic stem cells (hESCs) must be transformed into fate-restricted derivatives before use for cell therapy. Realizing the therapeutic potential of pluripotent hESC derivatives demands a better understanding of how a pluripotent cell becomes progressively constrained in its fate options to the lineages of tissue or organ in need of repair. Discerning the intrinsic plasticity and regenerative potential of human stem cell populations reside in chromatin modifications that shape the respective epigenomes of their derivation routes. The broad potential of pluripotent hESCs is defined by an epigenome constituted of open conformation of chromatin mediated by a pattern of Oct-4 global distribution that corresponds genome-wide closely with those of active chroma tin modifications. Dynamic alterations in chromatin states correlate with loss-of-Oct4-associated hESC differentiation. The epigenomic transition from pluripotence to restriction in lineage choices is characterized by genome-wide increases in histone H3K9 methylation that mediates global chromatin-silencing and somatic identity. Human stem cell derivatives retain more open epigenomic landscape, therefore, more developmental potential for scale-up regeneration, when derived from the hESCs ***in vitro*** **than from the CNS tissue** ***in vivo***. Recent technology breakthrough enables direct conversion of pluripotent hESCs by small molecule induction into a large supply of lineage-specific neuronal cells or heart muscle cells with adequate capacity to regenerate neurons and contractile heart muscles for developing safe and effective stem cell therapies. Nuclear translocation of NAD-dependent histone deacetylase SIRT1 and global chromatin silencing lead to hESC cardiac fate determination, while silencing of pluripotence-associated hsa-miR-302 family and drastic up-regulation of neuroectodermal Hox miRNA hsa-miR-10 family lead to hESC neural fate determination. These recent studies place global chromatin dynamics as central to tracking the normal pluripotence and lineage progres sion of hESCs. Embedding lineage-specific genetic and epigenetic developmental programs into the open epigenomic landscape of pluripotent hESCs offers a new repository of human stem cell therapy derivatives for the future of regenerative medicine.

## 1. INTRODUCTION

Human stem cells, both embryonic and somatic, hold great potential for cell replacement and regeneration therapies for human diseases. Gene expression analysis has indicated that stem cells do not seem to have a common core transcription profile that dictates the undifferentiated self-renewing state [1-4]; which suggests that gene expression alone is not sufficient to define either plasticity or lineage specification [5-8]. A search for a common set of transcribed genes that defines the characters of all stem cell derivatives, known as stemness, has been unsuccessful; there is virtually no overlap in the gene expression profiles of various types or derivations of stem cells, in spite of their apparent phenotypic similarity [8-13]. There exist overlaps in gene expression between cells of varying lineages yet a lack of overlap in phenotypes that ostensibly seem similar. Even the expression of a lineage-defining gene within stem cells seems to require additional epigenetic cues [14,15]. It is clear that epigenetic processes are providing additional regulatory dimensions to stem cell behavior [5-8].

The eukaryotic genome is packaged into chromatin, a nucleoprotein complex in which the DNA helix is wrapped around an octamer of core histone proteins to form a nucleosomal DNA structure, known as nucleosome, that is further folded into higher-order chromatin structures with the involvement of other chromosomal proteins [16-19]. Chromatin modifications, such as DNA methylation and histone modifications, serve as important epigenetic marks for active and inactive chromatin states, thus the principal epigenetic mechanism in early embryogenesis [20,21]. Regulation of chromatin structure by covalent modification of DNA and histones, by ATP-driven chromatin remodeling, and by incorporation of alternative histone variants can influence a broad range of cellular processes that include transcription, replication, recombination, and DNA repair; therefore, chromatin modifications have been implicated in a broad range of developmental processes [22,23]. Chromatin modification creates molecular landmarks that establish and maintain stage-specific gene expression patterns and global gene silencing during mammalian development. The activities of chromatin modification might be targeted to a specific gene through a sequence-specific DNA binding factor, which results in a cascade of chromatin regulation events that determine the fine tuning of cellular signaling and ultimately cell-fate choices. Therefore, regulation of chromatin-mediated lineage specification has become a fundamental mechanism in human stem cell lineage commitment and differentiation. However, these processes in human stem cell development, which may involve dynamic equilibrium between active and inactive chromatin states and establishment of chromatin codes by covalent modific ations on histones and DNA, remain to be understood.

The growing number of identified stem cell derivatives and escalating concerns for safety and efficacy of these cells towards clinical applications have made it increasingly crucial to assess the relative risk-benefit ratio of a stem cell to addressing a particular disease [5-8]. Discerning the intrinsic plasticity and regenerative potential of human stem cell populations reside in chromatin modifications that shape the respective epigenomes of their derivation routes [5-8]. Chromatin states have been used to characterize and compare the intricate plasticity and potential of stem cell populations [5-8]. Derivation of human embryonic stem cells (hESCs) provides a powerful *in vitro* model system to investigate molecular controls in human embryogenesis as well as an unlimited source to generate the diversity of human somatic cell types for regenerative medicine [24-26]. Pluripotent hESCs have both the unconstrained capacity for long-term stable undifferentiated growth in culture and the intrinsic potential for differentiation into all somatic cell types in the human body [24-26]. However, realizing the developmental and therapeutic potential of hESC derivatives has been hindered by the inefficiency and instability of generating clinically-relevant functional cells from pluripotent cells through conventional uncontrollable and incomplete multi-lineage differentiation [24,25]. Without a practical strategy to convert pluripotent cells direct into a specific lineage, previous studies and profiling of hESCs and their differentiating multi-lineage aggregates have compromised implications to molecular controls in human embryonic development [27-30]. Developing novel strategies for well-controlled efficiently directing pluripotent hESCs exclusively and uniformly towards clinically-relevant cell types in a lineage-specific manner is not only crucial for unveiling the molecular and cellular cues that direct human embryogenesis, but also vital to harnessing the power of hESC biology for tissue engineering and cell-based therapies.

To date, the lack of a clinically-suitable source of engraftable human stem/progenitor cells with adequate neurogenic potential has been the major setback in developing safe and effective cell-based therapies for regenerating the damaged or lost central nervous system (CNS) structure and circuitry in a wide range of neurological disorders. Similarly, the lack of a clinically-suitable human cardiomyocyte source with adequate myocardium regenerative potential has been the major setback in regenerating the damaged human heart. Given the limited capacity of the CNS and heart for self-repair, transplantation of hESC neuronal and heart cell therapy derivatives holds enormous potential in cell replacement therapy. There is a large unmet healthcare need to develop hESC-based therapeutic solutions to provide optimal regeneration and reconstruction treatment options for normal tissue and function restoration in many major health problems. However, realizing the developmental and therapeutic potential of hESC derivatives has been hindered by conventional approaches for generating functional cells from pluripotent cells through uncontrollable, incomplete, and inefficient multi-lineage differentiation [24-30]. Growing evidences indicate that incomplete lineage specification of pluripotent cells via multi-lineage differentiation often resulted in poor performance of such stem cell derivatives and/or tissue-engineering constructs following transplantation [24,25,31]. The development of better differentiation strategies that permit to channel the wide differentiation potential of pluripotent hESCs efficiently and predictably to desired phenotypes is vital for realizing the therapeutic potential of pluripotent hESCs.

The pluripotent hESC itself cannot be used for therapeutic applications. It has been recognized that pluripotent hESCs must be transformed into fate-restricted derivatives before use for cell therapy [7]. Realizing the therapeutic potential of pluripotent hESC derivatives demands a better understanding of how a pluripotent cell becomes progressively constrained in its fate options to the lineages of tissue or organ in need of repair [7]. Recent advances and breakthroughs in hESC research have overcome some major obstacles in bringing hESC therapy derivatives towards clinical applications, including establishing defined culture systems for *de novo* derivation and maintenance of clinical-grade pluripotent hESCs and lineage-specific differentiation of pluripotent hESCs by small molecule induction [5-8,25,32-36]. This technology breakthrough enables direct conversion of pluripotent hESCs into a large supply of high purity neuronal cells or heart muscle cells with adequate capacity to regenerate CNS neurons and contractile heart muscles for developing safe and effective stem cell therapies [5-8,25,32-36]. Transforming pluripotent hESCs into fate-restricted therapy derivatives dramatically increases the clinical efficacy of graft-dependent repair and safety of hESC-derived cellular products. Such milestone advances and medical innovations in hESC research allow generation of a large supply of clinical-grade hESC therapy derivatives targeting for major health problems. Currently, these hESC neuronal and cardiomyocyte therapy derivatives are the only available human cell sources with adequate capacity to regenerate neurons and contractile heart muscles, vital for CNS and heart repair in the clinical setting.

The pluripotence of hESCs that display normal stable expansion is associated with a globally active acetylated chromatin, as evident by high levels of expression and nuclear localization of active chromatin remodeling factors; weak expression or cytoplasmic localization of repressive chromatin remodeling factors that are implicated in transcriptional silencing; and residual H3 K9 methylation [5,37]. Profiling of chromatin modifications that make up the epigenome of pluripotent hESCs indicated that the broad potential of pluripotent hESCs is defined by an epigenome constituted of open conformation of chromatin mediated by a pattern of Oct-4 global distribution that corresponds genome-wide closely with those of active chromatin modifications [5,8]. Dynamic alterations in chromatin states correlate with loss-of-Oct4-associated hESC differentiation [5]. The epigenomic transition from pluripotence to restriction in lineage choices is characterized by genome-wide increases in histone H3K9 methylation that mediates global chromatin-silencing and somatic identity [5-8]. The intrinsic plasticity and regenerative potential of human stem cell derivatives can be differentiated by their epigenomic landscape features, and that human stem cell derivatives retain more open epigenomic landscape, therefore, more developmental potential for scale-up regeneration, when derived from the hESCs *in vitro* than from the CNS tissue *in vivo* [7,8]. Having achieved uniformly conversion of pluripotent hESCs to a cardiac or neural lineage with small molecule induction, in our recent reports, we further profiled chromatin modifications and microRNA (miRNA) expression in order to uncover the genetic and epigenetic mechanisms governing early lineage specification direct from the pluripotent stage [6-8,34]. Nuclear translocation of NAD (nicotinamide adenine dinucleotide)-dependent histone deacetylase SIRT1 and global chromatin silencing lead to hESC cardiac fate determination, while silencing of pluripotence-associated hsa-miR-302 family and drastic up-regulation of neuroectodermal Hox miRNA hsa-miR-10 family lead to hESC neural fate determination [6]. These recent studies place global chromatin dynamics as central to tracking the normal pluripotence and lineage progression of hESCs. Embedding lineage-specific genetic and epigenetic programs into the open epigenomic landscape of pluripotent hESCs offers a new dimension for direct control and modulation of hESC pluripotent fate when deriving clinically-relevant lineages for regenerative therapies.

## 2. EPIGENETIC MODIFICATIONS IN STEM CELL DEVELOPMENT

### 2.1 Chromatin Modification in the Establishment of Epigenetic Marks

Lineage-specific differentiation is a complex process that it is better characterized by the establishment of epigenetic marks than by specific gene activation. Recent studies indicate that epigenetic controls in stem cell fate decisions hold the key to some of the pressing questions regarding the underlying mechanisms of their developmental potential [5-8]. The development of chromatin/nucleosome-immunoprecipitation-coupled DNA microarray analysis (ChIP/NuIP-chip) and chromatin-immunoprecipitation-combined second-generation high-throughput sequencing (ChIP-seq) has provided the technology foundation for genome-wide approaches to profile alterations in spatial and temporal patterns of the developmental associated epigenetic markers in high-resolution [8,27,28,30,38-40]. Studies of chromatin modifications at a genome-wide scale have led to great advances in our understanding of the global phenomena of multiple epigenomes of human stem derivatives originated from embryos or various tissue types and developmental stages [8]. Large-scale profiling of developmental regulators and histone modifications has been used to identify epigenetic patterns for defining the phenotypic features of hESCs and their derivatives [8,27,28,30,41-42]. Mapping global patterns of chromatin dynamics in human stem cell derivatives will identify underlying molecular mechanisms as well as provide reliably predictive molecular parameters for comparing their intrinsic plasticity dominating stem cell behavior prior to transplantation [5-8]. Although genome-wide mapping of histone modifications and chromatin-associated proteins have already begun to reveal the mechanisms in mouse embryonic stem cell (ESC) differentiation [43], similar studies in hESCs are currently lacking due to the difficulty of conventional multi-lineage differentiation approaches in obtaining the large number of purified cells, particularly neurons and cardiomyocytes, typically required for ChIP-chip and ChIP-seq experiments [28,30,40].

### 2.2 Histone Methylation

**Fig. 1:**
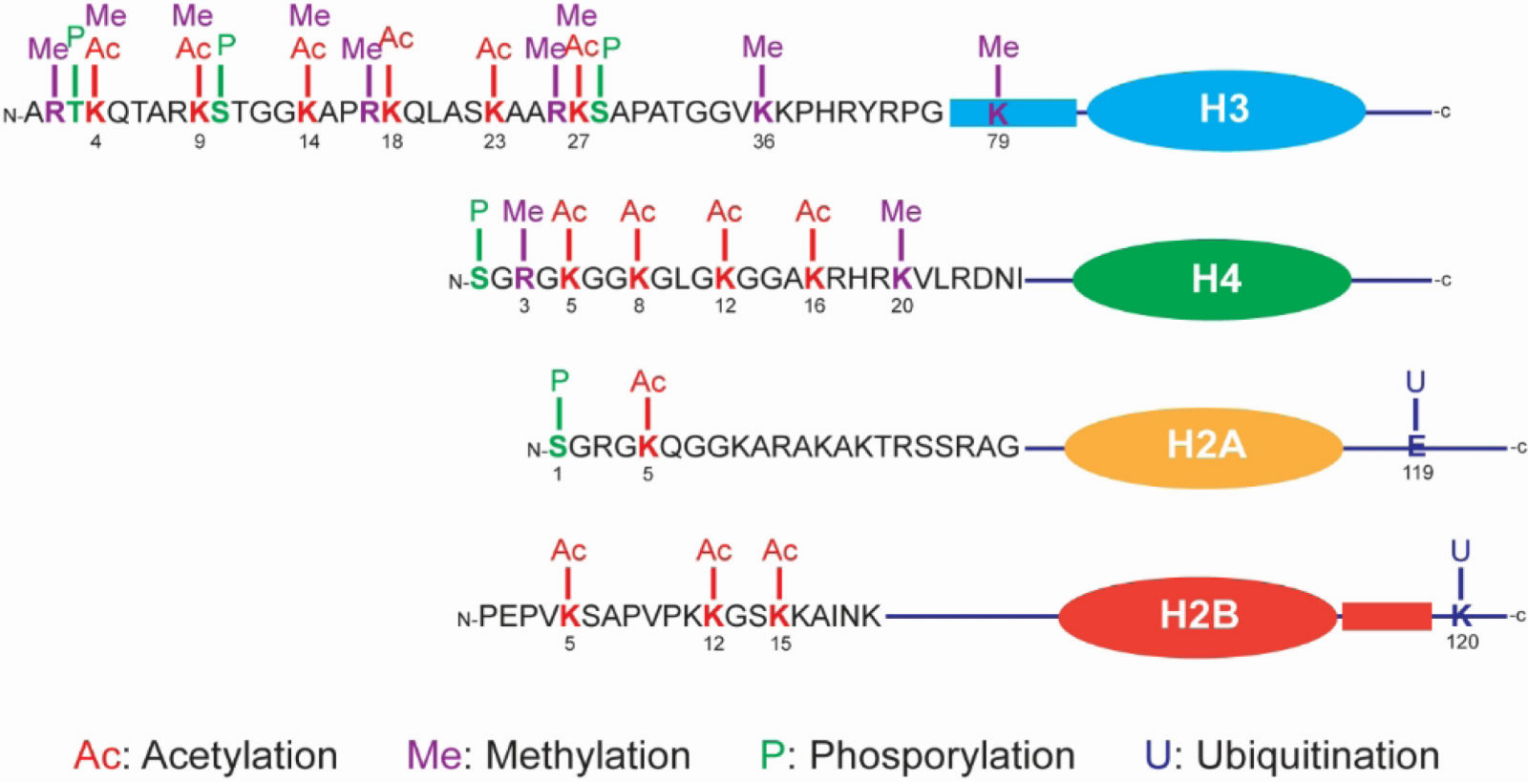
Chromatin modifications in histone tails Covalent histone modification is a highly regulated process and directly linked to diverse biological functions, such as transcription regulation, cell cycle progress, and genomic imprinting. Histones are small highly conserved basic proteins. Histone modifications include acetylation, deacetylation, methylation, phosphorylation, and ubiquitylation; and mostly occur in the N-terminal tails that are highly ***K*** *and* ***R*** *rich.*

Chromatin modification includes processes such as DNA methylation and histone acetylation, deacetylation, methylation, phosphorylation and ubiquitylation [16-19] (Fig. 1). These processes function cooperatively to establish and maintain active or inactive chromatin states in cellular development. Chromatin remodeling enzymes are largely involved in the control of cellular differentiation, and loss or gain of function is often correlated with pathological events [44]. Modification of core histone tails is far more complex than DNA methylation and involves many different histone modification enzymes (Fig. 1). Histones are small, highly conserved basic proteins. Local changes of chromatin architecture can be achieved by post-translational modifications of histones such as methylation, acetylation, phosphorylation, ubiquitination, sumoylation, and ADP-ribosylation [19,44,45].

These epigenomic changes are dynamic and allow for rapid repression or de-repression of specific target genes. In general, acetylation of core histones and methylation of K4 of histone H3 (H3K4me) correlate with transcriptional active (open) chromatin state, whereas deacetylation of core histones and methylation of K9 of histone H3 (H3K9me) correlate with transcriptional repressed (closed) chromatin state [16-19,46,47]. Previous reports have linked histone H3 K27 methylation to the repression of a special set of developmental genes in murine and human ESCs and H3 arginine methylation to the pluripotent inner cell mass (ICM) development in mouse embryos [27, 48-50]. The bivalent histone methylation marks include the H3K4me3 activation and the H3K27me3 repressive modifications confined to ESCs [27]. Several histone methyltransferases (HMT), including a histone H3 K4 methyltransferase and five histone H3 K9 methyltransferases such as SUV39H1, SUV39H2, G9a, ESET/SetDB1, and Eu-HMTaseI, have been identified in mammals [21]. Evidences indicate that SUV39H functions to methylates histones in heterochromatin, while G9a methylates histones in euchromatin which is essential for early embryogenesis [51,52]. Histone H3 K9 trimethylation by HMT has been shown as a mark for subsequent DNA methylation, suggesting the critical role of chromatin language in cellular development [45]. Enhancer of Zeste homlog 2 (EZH2), the HMT within Polycomb repressive II complexes, is essential for not only methylation of histone H3 on Lys 27 (H3K27me3) but also interaction with and recruiting DNA methyltransferases to methylate CpG at certain EZH2 target genes to establish firm repressive chromatin structures, contributing to tumor progression and the regulation of development and lineage commitment both in ESCs and adult stem cells [53-55]. In addition to being involved in Hox gene silencing, the EZH2/Polycomb complex and its associated HMT activity are important in biological processes including X-inactivation, germline development, stem cell pluripotence, and cancer metastasis [53-55].

### 2.3 Histone Acetylation and Deacetylation

Studies of transcriptional regulation have revealed that many of the transcription coactivators, including Gcn5, p300/CBP, PCAF, Tip60, and nuclear hormone receptor coactivators such as SRC-1, ACTR, and TIF2, as well as several subunits associated with RNA Polymerase II such as TAF(II) 250, TFIIIC and Elp3, contain intrinsic histone acetyltransferase (HAT) activity [56]. Acetylation impacts chromatin structure through the neutralization of the charge inherent to the amino group of lysine, thereby weakening intra-and inter-nucleosomal interactions of the chromatin fiber and facilitating its decondensation by increasing accessibility to the nucleosomal DNA [56]. In addition, acetylation is recognized or targeted by the bromodomain of a variety of chromatin factors that mediates transcription activation and ATP-dependent chromatin remodeling through recruitments of other specific regulators [56].

On the other hand, histone deacetylases (HDAC) are associated with global transcription corepressor such as Sin3 and NcoR/SMRT [57-59]. Inhibitors of HDACs have been found to cause stem cell differentiation as well as growth arrest, differentiation, and apoptosis of many tumor cells [5,60]. Three classes of HDACs have been identified so far. Class I human HDACs include HDAC1, 2, 3, and 8, and are homologous to yeast Rpd3 [17]. Class II HDACs, which are expressed in specific tissues, contain a group of large molecules and are homologous to yeast Hda1, such as HADC4, 5, 6, 7, and 9 [17]. Class III HDACs consist of a group of NAD-dependent histone deacetylases known as Sirtuin and are homologous to yeast Sir2 (silent information regulator 2) that is involved in transcriptional silencing [19]. Sir2, the yeast longevity and transcriptional silencing protein, is recruited to DNA by chromatin binding factors to spread to the entire locus and mediate gene silencing during mating cell type switch [61]. Sir2 in higher organisms plays an essential role in heterochromatic silencing and euchromatic repression, and associates with the bHLH repressor proteins, the key regulators of development [62, 63]. Analysis of null mutant of Sir2 in mouse suggests that mammalian Sir2 has an essential role in embryogenesis and gametogenesis [64]. *In vitro* reconstitution studies indicate that histone deacetylation by Sir2 generates a conformational change or rearrangement of histones into a transcriptionally repressive chromatin structure [19]. Sir2 human orthologue SIRT1 physically associates with DNA cytosine methyltransferase Dnmt1 and can deacetylate acetylated Dnmt1 *in vitro* and *in vivo*, which has different effects on the functions of Dnmt1 dependent on the lysine residues [65]. Interestingly, studying of transcriptional silencing in yeast also revealed several members of MYST family of HATs such as Sas2 (something about silencing), Sas3, and Esa1 (essential Sas2-related acetyltransferase) are also involved in gene silencing [56]. Human homologues of MYST family of acetyltransferases include the Tip60 (Tat interactive protein 60), MOZ (monocytic leukemia zinc finger protein), MORF (MOZ-related factor), and HBO1 (histone acetyltransferase bound to ORC). Tip60/p400 complexes have been described as regulating mouse ESC gene expression via Nanog and H3K4 methylation [66]. SIRT1 negatively regulates the activities, functions, and protein levels of hMOF and TIP60 [67]. That pools of HAT and HDAC are so evolutionally conserved suggests that a mechanism similar to the chromatin-mediated cell type switch in yeast may contribute to lineage-specification in human stem cell development.

### 2.4 Chromatin Remodeling Factors

Chromatin remodeling factors are ATP-utilizing motor proteins that mediate the interaction of proteins with nucleosomal DNA by DNA/nucleosome-translocation [16,68]. ATP-dependent nucleosomal remodeling factor hBrm and hBrg1 (components of hSWI/SNF chromatin remodeling complex), and hSNF2H (human homolog of ISWI [Imitation Switch], a component of hACF/WCRF and hRSF chromatin remodeling complexes) have been shown to be involved in cellular functions such as chromatin assembly, chromosome structure, global remodeling of nuclei, DNA replication, recombination, and repair [16,69]. Two murine members of ISWI, SNF2H and SNF2I, display distinct differential expression patterns in the brain: SNF2H is prevalent in proliferating cell populations, whereas, SNF2I is predominantly expressed in terminally differentiated neurons after birth [70]. Various previous reports in flies and mouse show the involvement of chromatin remodeling factors, such as Brm (an ATP-dependent chromatin-remodeling factor implicated in mediating H3K9 methylation) and REST/NRSF (a HDAC2-associated transcriptional repressor complex), in neuronal development and function [71-76]. Chromatin remodeling complexes with ubiquitous subunits including two ATPases Brg1 and Brm and HDACs have been shown to mediate repression of neuronal-specific genes [72,76-78]. Smyd1/Bop (SET and MYND domain containing 1) and members of Class II HDACs (HDAC 5, 7, 9) are involved in regulating the development of mouse ventricular cardiomyocytes [79-82]. A muscle-specific member of the SWI/SNF complex, BAF60c (BRG1/BRM-associated factor 60 c), is essential to activate both skeletal and cardiac muscle programs in mouse [83,84]. Another example of a chromatin remodeling factor is Ikaros, a sequence-specific DNA-binding zinc finger protein and an integral component of nucleosomal remodeling and deacetylation complexes (NURD) that contains chromatin remodeling factor Mi-2, HDAC1 and HDAC2 [69,85]. Deregulation of Ikaros has been found to induce leukemia, indicating it is an essential regulator of lymphocyte development [22]. Brg1 has been shown to interact with the key regulators of pluripotence, Oct4, Sox2, and NANOG, and exhibit a highly correlated genome-wide binding patterns with these proteins in mouse ESCs [86,87], suggesting a cooperative role of SWI/SNF complexes in keeping the cells in the undifferentiated state [69,88]. In addition, chromodomain helicase DNA binding proteins (CHD), which contain two chromodomains, hence exhibiting high affinity for methylated histones, especially H3K4me2/3, appear to be required for maintaining a open chromatin conformation in mouse ESCs [69,89]. However, the precise functions of those chromatin remodeling factors in regulation of hESC pluripotence and different iation remain to be shown.

### 2.5 DNA Methylation

DNA methylation patterns are established and maintained by DNA methyltransferases. Methyl-CpG-binding proteins (MBD) are believed to then recruit NURD that contain Mi-2 (an ATP-dependent chromatin remodeling factor) and HDAC to deacetylate histones and induce gene silencing [90-93]. In addition, methylation of histone H3 K9 by HMT can trigger the binding of heterochromatin protein 1 (HP1) to methylated histones, which might in turn recruit DNA methyltransferases to stabilize the inactive chromatin [94]. Five methylated DNA binding proteins (MeCP2, MBD1, MBD2, MBD3, and MBD4) and three active DNA cytosine methyltransferases (Dnmt1, Dnmt3a, and Dnmt3b) have been identified in mammals. Dmnt1 is a ubiquitously expressed maintenance methyltransferase and functions to restore DNA methylation patterns after DNA replication, while Dnmt3a and Dnmt3b function to initiate *de novo* methylation and establish new DNA methylation patterns during development [95]. Targeted deletions of Dmnt1 and MBD3 in mice are embryonic lethal [95,96], while mutations in MeCP2 result in deficiency in neural development and cause mental retardation (Rett syndrome) [97-101]. Mouse adult neural stem cells (NSCs) lacking MBD1 have also been shown deficiency in neural development [102]. The deficiency of MBD3 leads to hyperacetylation and loss of mouse ESC pluripotence [103].

DNA methylation and demethylation play an important role in ESC development as well as somatic cell reprogramming and imprint erasure [69,104-107]. DNA methylation is essential for normal development and has been implicated in many pathologies including cancer [107]. Methylation of CpGs establishes dynamic epigenetic marks that undergo extensive changes during cellular differentiation, particularly in regulatory regions outside of core promoters [107]. Genome-wide mapping of DNA methylation patterns at proximal promoter regions in mouse ESCs suggested that most methylated genes are differentiation associated and repressed, while the unmethylated gene set includes many housekeeping and pluripotence genes [108]. Somatic cell nuclear transfer and transcription-factor-based reprogramming have been used to revert adult cells to an embryonic-like state with extremely low efficiencies [109-112]. Pluripotence-inducing factors, most of which are known oncogenes, have been used to reprogram mouse and human somatic cells to induced pluripotent stem cells (iPS cells) [109-112]. DNA hydroxylase Ten-Eleven Translocation (Tet) family of enzymes, which convert 5-methylcytosine (5mC) to 5-hydroxymethylcytosine (5hmC) in various embryonic and adult tissues, facilitates mouse pluripotent stem cell induction by promoting Oct4 demethylation and reactivation [105]. The 5hmC enrichment is involved in the demethylation and reactivation of genes and regulatory regions that are important for pluripotence [105]. However, factor-based reprogramming can leave an epigenetic memory of the tissue of origin that may influence efforts at directed differentiation for applications in disease modelling or treatment and is even less effective at establishing the ground state of pluripotence than that of somatic nuclear transfer [113]. Somatic cell nuclear transfer and factor-based reprogramming are incapable of restoring a correctepigenetic pattern of pluripotent ESCs, which accounts for abnormal gene expression, accelerated senescence, and immune-rejection following transplantation of reprogrammedcells [114-116]. These major drawbacks have severely impaired the utility of reprogrammed or deprogrammed or direct differentiated somatic cells as viable therapeutic approaches.

These evidences suggest that a chromatin-mediated mechanism may be central to understanding how potential of a stem cell is restricted such that a particular phenotype emerges and, hence, central to judging the plasticity and commitment of a human stem cell. Although it is evident that chromatin modification plays a crucial role in epigenetic programming in human embryogenesis, the molecular mechanism involved is largely unknown. It is known that undifferentiated hESCs express a unique group of genes, including Oct-4, as well as possess specific enzymatic activities such as alkaline phosphatase and telomerase [26]. However, none of these markers, in isolation, is exclusively expressed by undifferentiated hESCs. Rather, their presence as a group is associated with the undifferentiated state [26, 117]. Plasticity and the pre-differentiation state remain poorly understood at the molecular level. For mouse ESCs, two independent regulatory pathways, the cytokine-dependent LIF/gp130/Stat3 pathway and the cytokine-independent pathway mediated by the homeoprotein Nanog are required for the maintenance of pluripotence and self-renewal [118,119]. Both pathways require the sustained expression of Oct-4. However, the molecular regulation mechanism is different in mouse and human. Human and mouse ESCs actually express opposite markers and require distinct conditions for maintenance and differentiation [25,120,121]. Unlike mouse ESCs, the maintenance of undifferentiated hESCs does not require LIF and the LIF/Stat3 signaling pathway, suggesting that an entirely different regulatory system might be employed in human [122,123]. In embryogenesis, only cells in the ICM express Oct-4. Loss of Oct-4 at the blastocyst stage causes these cells to differentiate into trophectoderm, while Oct-4 expression ensures embryonic germ layer assignment and lineage differentiation [117]. The restriction of Oct-4 expression *in vivo* and *in vitro* appears more likely to result from establishment of a general active chromatin state rather than an outcome of specific activators [117]. Investigating epigenetic controls in human stem cell plasticity, potency, and fate decisions may unravel the critical regulatory dimension and definition regarding how hESCs maintain self-renewal and prevent differentiation as well as how to direct lineage-specific differentiation of hESCs.

## 3. THE PLURIPOTENCE OF HESCS CONFORMS TO A GLOBALLY ACTIVE HIGHLY ACCESSIBLE EPIGENOME OPEN FOR ENDLESS POSSIBILITY IN HUMAN DEVELOPMENT

### 3.1 The Normality and Positivity of hESC Open Epigenome Distinguish Pluripotent hESCs from iPS Cells and Tissue-Resident Stem Cells

Pluripotent hESCs, derived from the pluripotent ICM or epiblast of the human blastocyst, have both the unconstrained capacity for long-term stable undifferentiated growth in culture and the intrinsic potential for differentiation into all somatic cell types in the human body, holding tremendous potential for restoring human tissue and organ function. The hESCs are not only pluripotent, but also incredibly stable and positive, as evident by that only the positive active chromatin remodeling factors, but not the negative repressive chromatin remodeling factors, can be found in the pluripotent epigenome of hESCs [5-8,37]. The normality and positivity of hESC open epigenome also differentiate pluripotent hESCs from any other stem cells, such as the pluripotent iPS cells reprogrammed from adult cells and the tissue-resident stem cells [5-8]. Although pluripotent, the iPS cells are made from adult cells, therefore, iPS cells carry many negative repressive chromatin remodeling factors and unerasable genetic imprints of adult cells that pluripotent hESCs do not have [104-106,113,114]. The traditional sources of engraftable human stem cells with neural potential for transplantation therapies have been multipotent human neural stem cells (hNSCs) isolated directly from the human fetal neuroectoderm or CNS [24,25,124-128]. Despite some beneficial outcomes, CNS-derived hNSCs appeared to exert their therapeutic effect primarily by their non-neuronal progenies through producing trophic and/or neuro-protective molecules to rescue endogenous host neurons, but not related to regeneration from the graft [24,126,127]. Compared to hESCs and their neural derivatives, the epigenome of tissue-resident CNS-derived hNSCs is more deacetylated, methylated, and compacted as a result of global increases in histone H3K9 methylation mediated repressive chromatin remodeling, therefore, stem cells derived from tissues have acquired more silenced chromatin and are likely resides at a more advanced stage of development with more limited developmental potential and declining plasticity with aging for regeneration [7,8]. So far, due to these major limitations in their intrinsic plasticity and regenerative potential, cell therapies based on CNS-derived hNSCs have not yielded the satisfactory results expected for clinical trials to move forward [129]. Therefore, the intrinsic plasticity and regenerative potential of human stem cell derivatives can be differentiated by their epigenomic landscape features, and that human stem cell derivatives retain more open epigenomic landscape, therefore, more developmental potential and plasticity for scale-up regeneration, when derived from the hESCs *in vitro* than from the CNS tissue *in vivo* [7,8].

### 3.2 The Pluripotence of hESCs is Enabled by a Globally Acetylated Open Chromatin

**Fig. 2:**
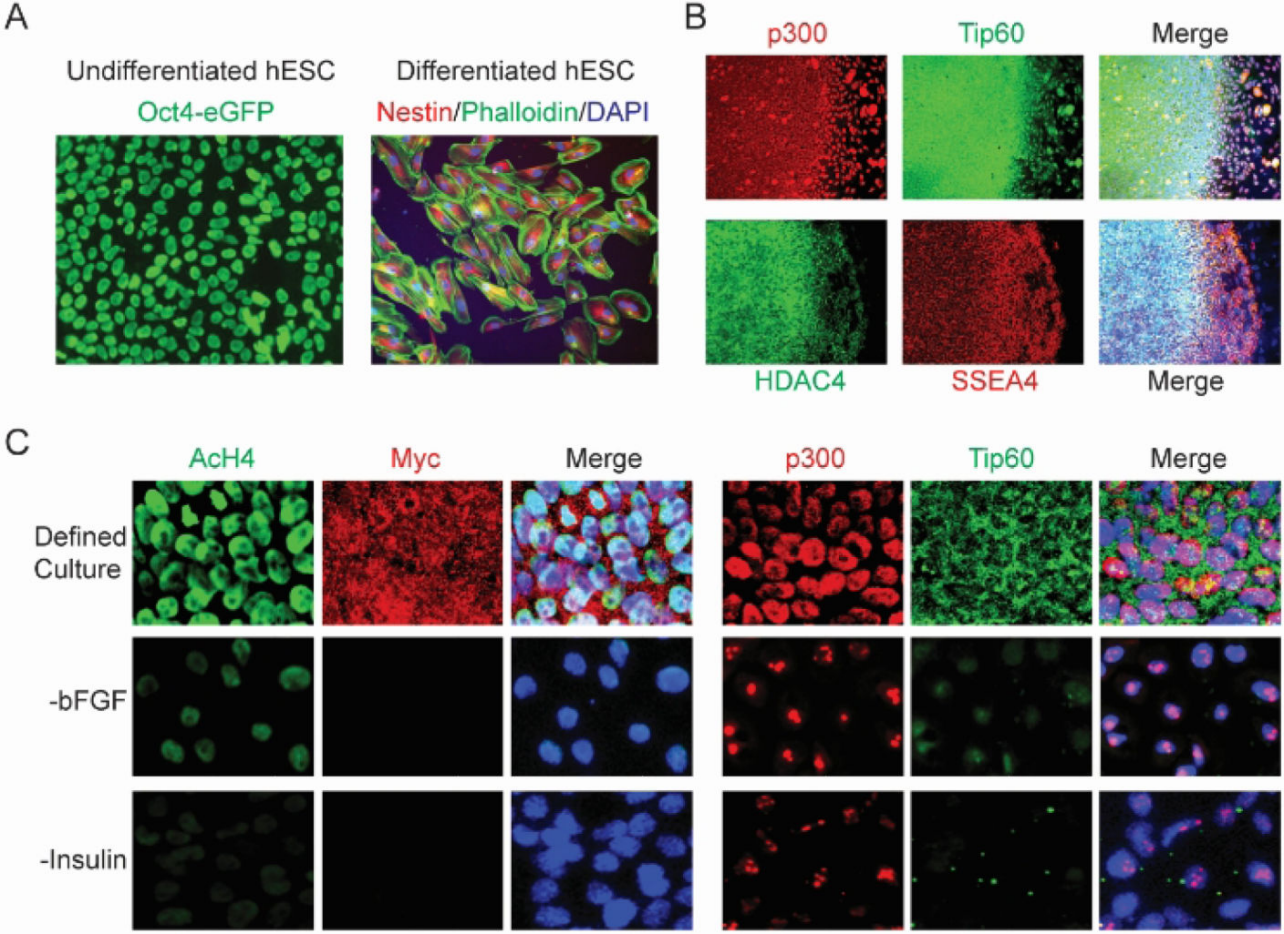
The pluripotent state of hESCs is associated with active chromatin remodeling **(A).**. Undifferentiated hESCs carrying Oct-4-driven eGFP (green) express Oct-4 and differentiated hESCs after treated with HADC inhibitor TSA express Nestin (red) and phalloidin (green). **(B).**. Undifferentiated hESC colonies, as indicated by SSEA-4 expression (red), express nuclear localized p300 (red) and cytoplasmic localized Tip60 (green) and HADC4 (green). **(C).** Undifferentiated hESCs maintained under the defined culture in the presence of bFGF and insulin have a heavily acetylated chromatin as suggested by strong immunopositivity to acetylated histone H4 (AcH4, green), Myc (red), and HATs Tip60 (green) and p300 (red). When either bFGF or insulin is omitted, the differentiated cells show significantly reduced immunoreactivity to AcH4, Myc, Tip60, and nuclear focal localization of p300. All cells are indicated by DAPI staining of their nuclei (blue).

The pluripotence of hESCs that display normal stable expansion is enabled by a globally acetylated, decondensed, highly accessible chromatin associated with high levels of expression and nuclear localization of active chromatin remodeling factors that include acetylated histone H3 and H4 (acH3 and acH4); the active ATP-dependent chromatin-remodeling factor Brg-1 and hSNF2H; HAT p300; and the class I basal transcription maintenance HDAC1 [5-8] (Fig. 2). The association of pluripotence of hESCs with a globally open chromatin state conforms to highly dynamic active epigenomic remodeling, which provides the molecular foundation for the normal stable pluripotence of hESCs [5,8]. By contrast, those repressive chromatin remodeling factors that are implicated in transcriptional silencing, including repressive chromatin-remodeling factor Brm and Mi-2 involved in histone H3 K9 methylation or nucleosome deacetylation of NURD; HAT PCAF, Tip60, Moz, and HBO-1; tissue-specific class II HDAC4, 5, 6, 7; the class III NAD-dependent HDAC SIRT1; and the H3 K9 HMT SUV39H1, were either weakly expressed or localized to cytoplasm and/or cell surface, indicating that they are mostly inactive in maintaining the pluripotent epigenome of hESCs [5] (Fig. 2). Although undifferentiated hESCs display the bivalent histone marks that include the H3K4me3 activation and the H3K27me3 repressive modifications, only residual nucleosomal H3 K9 methylation, a chromatin modification implicated in transcriptional repression during development, was observed in the pluripotent epigenome of hESCs [5,8,27,69]. Residual repressive chromatin remodeling implicated in chromatin silencing and transcriptional repression might be essential for stabilizing the pluripotent state of hESCs with a globally active open epigenome at a normal developmental stage [5]. In fact, aberrant H3 K9 methylation at embryonic stage has been associated with DNA hypermethylation and cell malignant transformation in abnormal pluripotent embryonic carcinoma cells [130,131].

Genome-wide profiling of chromatin modifications that make up the epigenome of pluripotent hESCs indicated that the broad potential of pluripotent hESCs is defined by an epigenome constituted of open conformation of chromatin mediated by a pattern of Oct-4 global distribution that corresponds genome-wide closely with those of active chromatin modifications, as marked by either acetylated histone H3 or H4 [8]. Profiling of Oct-4 binding by genome-wide approaches suggests that Oct-4 binding is widespread and particularly enriched for upstream and downstream of transcribed regions [8]. A considerable amount of evidence suggests that acetylation of histone H3 and H4 has distinct functional and temporal patterns [7,8,19,132]. The H3 modifications seem to be connected to proper control of gene expression, whereas acetylation of H4 seems to be most important in histone deposition and chromatin structure [7,8,19,132]. It appears that the overall pattern of deposition peaks of Oct-4 corresponds more closely with that of acetylated H4 than with that of acetylated H3 in general [8].

The wide distribution pattern of Oct-4 coincident with sites of active chromatin modification genome-wide suggested that Oct-4 might play an essential role in the interface of chromatin and transcription regulation to maintain a pluripotent epigenome enabled by a globally active open chromatin [5,8]. A dynamic progression from acetylated to transient hyperacetylated to hypoacetylated chromatin states correlates with loss-of-Oct4-associated hESC differentiation, further suggesting that Oct-4 might play an essential role in preserving the globally active chromatin state in pluripotent hESCs by maintaining a balanced level of histone acetylation and that changes in Oct-4 expression appeared to promote hESC differentiation by allowing alterations in chromatin state [5]. RNA interference directed against Oct-4 and HDAC inhibit or analysis support this pivotal link between chromatin dynamics and hESC differentiation [5] (Fig. 2). The epigenomic transition from pluripotence to restriction in lineage choices is characterized by genome-wide increases in histone H3K9 methylation that mediates global chromatin-silencing and somatic identity [5-8]. These recent studies reveal an epigenetic mechanism for placing global chromatin dynamics as central to tracking the normal pluripotence and lineage progression of pluripotent hESCs [5-8]. The transitions between distinct chromatin states, from the open acetylated chromatin of the pluripotent hESC to the more compact deacetylated and methylated chromatin of the differentiated cells or somatic tissue-resident cells, suggest a self-regulated complex dynamic determined by a progression of global chromatin remodeling as lineage commitment proceeds through the developmental processes [5-8].

## 4. EMBEDDING LINEAGE-SPECIFIC GENETIC AND EPIGENETIC DEVELOPMENTAL PROGRAMS INTO THE OPEN EPIGENOMIC LANDSCAPE OF PLURIPOTENT HESCS

### 4.1 Lineage-Specific Differentiation of Pluripotent hESCs by Small Molecule Induction Opens the Door to Investigate Molecular Embryogenesis in Human Development

Understanding the much more complex human embryonic development has been hindered by the restriction on human embryonic and fetal materials as well as the limited availability of human cell types and tissues for study. In particular, there is a fundamental gap in our knowledge regarding the molecular networks and pathways underlying the CNS and the heart formation in human embryonic development. The enormous diversity of human somatic cell types and the highest order of complexity of human genomes, cells, tissues, and organs among all the eukaryotes pose a big challenge for characterizing, identifying, and validating functional elements in human embryonic development in a comprehensive manner. Many of the biological pathways and mechanisms of lower-organism or animal model systems do not reflect the complexity of humans and have little implications for the prevention and cure of human diseases in the clinical setting. As a result of lacking a readily available human embryonic model system, the mainstream of biomedical sciences is becoming increasingly detached from its ultimate goal of improving human health. Derivation of hESCs provides not only a powerful *in vitro* model system for understanding human embryonic development, but also unique revenue for bringing the vast knowledge generated from the mainstream of biomedical sciences to clinical translation. Development and utilization of hESC models of human embryonic development will facilitate rapid progress in identification of molecular and genetic therapeutic targets for the prevention and treatment of human diseases. Such hESC research will dramatically increase the overall turnover of investments in biomedical sciences to optimal treatment options for a wide range of human diseases.

To overcome some of the major obstacles in basic biology and therapeutic application of hESCs, recent studies have resolved the elements of a defined culture system necessary and sufficient for sustaining the epiblast pluripotence of hESCs, serving as a platform for *de novo* derivation of animal-free therapeutically-suitable hESCs and well-controlled efficient specification of such pluripotent cells exclusively and uniformly towards a particular lineage by small molecule induction [5,25,32]. These recent reports show that pluripotent hESCs maintained under the defined culture conditions can be uniformly converted into a specific neural or cardiac lineage by small molecule induction [5-8,25,32-36]. Retinoic acid (RA) was identified as sufficient to induce the specification of neuroectoderm direct from the pluripotent state of hESCs and trigger a cascade of neuronal lineage-specific progression to human neuronal progenitors (hESC-I hNuP) and neurons (hESC-I hNu) of the developing CNS in high efficiency, purity, and neuronal lineage specificity by promoting nuclear translocation of the neuronal specific transcription factor Nurr-1 [6-8,25,34,35]. Unlike the two prototypical neuroepithelial-like Nestin-positive hNSCs derived from CNS *in vivo* or hESC *in vitro* via conventional multi-lineage differentiation, these *in vitro* neuroectoderm-derived Nurr1-positive hESC-I hNuPs did not express the canonical hNSC markers, but yielded neurons efficiently and exclusively, suggesting that they are a more neuronal lineage-specific embryonic neuronal progenitor than the prototypical neuroepithelial-like hNSCs [6-8,25,34,35]. Similarly, we found that such defined conditions rendered small molecule nicotinamide (NAM) sufficient to induce the specification of cardiomesoderm direct from the pluripotent state of hESCs by promoting the expression of the earliest cardiac-specific transcription factor Csx/Nkx2.5 and triggering progression to cardiac precursors and beating cardiomyocytes with high efficiently [6,25,32,36]. This technology breakthrough enables neuronal or cardiac lineage-specific differentiation direct from the pluripotent state of hESCs with small molecule induction, providing much-needed *in vitro* model systems for investigating molecular controls in human CNS or heart development in embryogenesis as well as a large supply of clinical-grade human neuronal or heart muscle cells across the spectrum of developmental stages for tissue engineering and cell therapies. It opens the door for further identification of genetic and epigenetic developmental programs underlying hESC neuronal or cardiomyocyte specification.

Large-scale profiling of developmental regulators and histone modifications by genome-wide approaches has been used to identify the developmental associated epigenetic markers in high-resolution, including in hESCs and their derivatives [8,27,28,30,41-42]. In addition, recently advances in human miRNA expression microarrays and ChIP-seq have provided powerful genome-wide, high-throughput, and high resolution techniques that lead to great advances in our understanding of the global phenomena of human developmental processes [6,30,34,40,133,134]. MiRNAs act as the governors of gene expression networks, thereby modify complex cellular phenotypes in development or disorders [135-137]. MiRNAs play a key role in regulation of ESC identity and cell lineage in mouse and human ESCs [133-136]. MiRNA expression profiling using microarrays is a powerful high-throughput tool capable of monitoring the regulatory networks of the entire genome and identifying functional elements in hESC development [6,34]. ChIP-seq is a most recently developed technique for genome-wide profiling of DNA-binding proteins, histone or nucleosome modifications using next-generation deep DNA sequencing technology [40,138,139]. ChIP-seq offers higher resolution, less noise and greater coverage than its array-based predecessor ChIP-chip, and has become an indispensable tool for studying gene regulation and epigenetic mechanisms in development [40,138,139]. ChIP-seq provides a means to rapidly determine the precise genomic location of transcription factor binding sites and histone modifications on a genome-wide scale. However, without a practical strategy to convert pluripotent cells direct into a specific lineage, previous studies are limited to profiling of hESCs differentiating multi-lineage aggregates, such as embryoid body (EB), that contain mixed cell types of endoderm, mesoderm, and ectoderm cells or a heterogeneous population of EB-derived cardiac or cardiovascular cells that contain mixed cell types of cardiomyocytes, smooth muscle cells, and endothelial cells [28-30]. Those previous reports have not achieved to utilize high-throughput approaches to profile one particular cell type differentiated from hESCs, such as cardiomyocytes [28-30]. Their findings have been limited to a small group of genes that have been identified previously, and thus, have not uncovered any new regulatory pathways unique to humans [28-30]. Due to the difficulty of conventional multi-lineage differentiation approaches in obtaining the large number of purified cells, particularly neurons and cardiomyocytes, typically required for ChIP and ChIP-seq experiments, studies to reveal the mechanism in hESC differentiation remain lacking [30,40]. Recent technology breakthrough in lineage-specific differentiation of pluripotent hESCs by small molecule direct induction allows generation of homogeneous populations of neural or cardiac cells direct from hESCs without going through the multi-lineage EB stage [5-8,25,32-36]. This novel small molecule direct induction approach renders a cascade of neural or cardiac lineage-specific progression directly from the pluripotent state of hESCs, providing much-needed *in vitro* model systems for investigating the genetic and epigenetic programs governing the human embryonic CNS or heart formation. Such *in vitro* hESC model systems enable direct generation of large numbers of high purity hESC neuronal or cardiomyocyte derivatives required for ChIP-seq analysis to reveal the mechanisms responsible for regulating the patterns of gene expression in hESC neuronal or cardiomyocyte specification. It opens the door for further characterizing, identifying, and validating functional elements during human embryonic neurogenesis or cardiogenesis in a comprehensive manner. Further using genome-wide approaches to study hESC models of human CNS or heart formation will not only provide missing knowledge regarding molecular human embryogenesis, but also lead to more optimal stem-cell-mediated therapeutic strategies for the prevention and treatment of CNS or heart diseases.

### 4.2 A Predominant Genetic Mechanism via Silencing of Pluripotence-Associated miRNAs and Drastic Up-Regulation of Neuroectodermal Hox miRNAs Governs hESC Neural Fate Determination

Having achieved uniformly conversion of pluripotent hESCs to a cardiac or neural lineage with small molecule induction, in our recent reports, we further profiled chromatin modifications and miRNA expression in order to uncover the genetic and epigenetic mechanisms governing hESC lineage specific progression direct from the pluripotent stage [6-8,34]. These *in vitro* neuroectoderm-derived Nurr1-positive hESC-I hNuPs expressed high levels of active chromatin modifiers, including acetylated histone H3 and H4, HDAC1, Brg-1, and hSNF2H, retaining an embryonic acetylated globally active chromatin state, which suggests that they are a more plastic human embryonic neuronal progenitor [7,8]. Consistent with this observation, several repressive chromatin remodeling factors regulating histone H3K9 methylation, including SIRT1, SUV39H1, and Brm, were inactive in hESC-I hNuPs [7,8]. To uncover key regulators, genome-scale profiling of miRNA differential expression patterns was used to identify novel sets of human development-initiating miRNAs upon small-molecule-induced neural and cardiac lineage specification direct from the pluripotent stage of hESCs [6]. A unique set of pluripotence-associated miRNAs was down-regulated, while novel sets of distinct cardiac-and neural-driving miRNAs were up-regulated upon the induction of hESC lineage specific differentiation [6]. The expression of pluripotence-associated hsa-miR-302 family was silenced and the expression of Hox miRNA hsa-miR-10 family that regulates gene expression predominantly in neuroectoderm was induced to high levels in these hESC-derived neuronal progenitors hESC-I hNuPs [6,34]. Following transplantation, they engrafted widely and yielded well-dispersed and well-integrated human neurons at a high prevalence within neurogenic regions of the brain, demonstrating their potential for neuron replacement therapy [7,34]. Genome-scale profiling of miRNA differential expression patterns during hESC neuronal lineage-specific progression further identified novel sets of stage-specific human embryonic neurogenic miRNAs, including silencing of the prominent pluripotence-associated hsa-miR-302 family and drastic expression increases of Hox hsa-miR-10 and the let-7 miRNAs [6,34]. These miRNA profiling studies suggested that distinct sets of stage-specific human embryonic neurogenic miRNAs, many of which were not previously linked to neuronal development and function, contribute to the development of neuronal identity in human CNS formation [6,34]. The miR-10 genes locate within the Hox clusters of developmental regulators and are coexpressed with a set of Hox genes to repress the translation of Hox transcripts [140]. The drastic expression increase of hsa-miR-10 upon exposure of hESCs to RA suggested that RA might induce the expression of Hox genes and co-expression of Hox miRNA hsa-miR-10 to silence pluripotence-associated genes and miRNA hsa-miR-302 to drive a neuroectoderm fate switch of pluripotent hESCs [6,34]. The evolutionarily conserved Hox family of homeodomain transcription factors plays fundamental roles in regulating cell fate specification to coordinate body patterning during development [141,142]. Coordination between genetic and epigenetic programs regulates cell fate determination in developmental processes [142]. Once established, Hox gene expression is maintained in the original pattern by Polycomb (PcG) and trithorax (trxG) group proteins that play essential roles in epigenetic developmental processes [141-144]. The PcG and trxG group complexes control the maintenance of Hox gene expression in appropriate domains by binding to specific regions of DNA and directing the posttranslational modification of histones to silence or activate gene expression [141-144].

Developing strategies for complex 3D multi-cellular models of human embryogenesis and organogenesis will provide a powerful tool that enables analysis under conditions that are tightly regulated and authentically representing the *in vivo* spatial and temporal patterns [25]. Therefore, as an authentic and reliable alternative to animal models, we combined our breakthrough in establishing hESC neuronal lineage-specific differentiation protocol with the advancements in 3D culture microenvironments to develop the multi-cellular 3D CNS model targeted for rapid and high fidelity safety and efficacy evaluation of therapeutic candidates and cell therapy products. Under 3D neuronal subtype specification conditions, these hESC-derived neuronal cells further proceeded to express subtype neuronal markers associated with ventrally-located neuronal populations, such as dopaminergic neurons and motor neurons [34], demonstrating their potential for neuron regeneration *in vivo* as stem cell therapy to be translated to patients in clinical trials. These recent studies suggest that these hESC neuronal derivatives have acquired a neuronal lineage-specific identity by silencing pluripotence-associated miRNAs and inducing the expression of miRNAs linked to regulating human CNS development to high levels, therefore, highly neurogenic *in vitro* and *in vivo* [6,7,34]. Novel lineage-specific differentiation approach by small molecule induction of pluripotent hESCs not only provides a model system for investigating human embryonic neurogenesis, but also dramatically increases the clinical efficacy of graft-dependent repair and safety of hESC-derived cellular products [6-8,25,34,35]. Thus, it offers a large supply of plastic human cell source with adequate capacity to regenerate the CNS neurons for CNS tissue engineering and developing safe and effective stem cell therapy to restore the normal nerve tissue and function.

### 4.3 A Predominant Epigenetic Mechanism via SIRT1-Mediated Global Chromatin Silencing Governs hESC Cardiac Fate Determination

A group of miRNAs displayed an expression pattern of up-regulation upon hESC cardiac induction by NAM, including the clusters of hsa-miR-1268, 574-5p, 92 family, 320 family, 1975, 1979, 103, and 107 [6]. Several groups identified miRNAs as the governors of gene expression in response to myocardial infarction (MI) and during post-MI remodeling of adult hearts [137]. Signature patterns of miRNAs identified that miR-1, 29, 30, 133, 150, and 320 were down-regulated, while miR-21, 23a, 125, 195, 199 and 214 were up-regulated during pathological cardiac remodeling in the adult hearts of rodents and humans [137]. Gain-and loss-of-function studies in mice revealed miR-1 and miR-133 as key regulators in cardiac development and stress-dependent remodeling, miR-138 in control of cardiac patterning, miR-143/145 and miR126 in cardiovascular development and angiogenesis [137]. The miR-1 and miR-133 were previously shown to promote mesoderm and muscle differentiation from mouse and human ESCs by repressing nonmuscle gene expression [135,137]. Recent miRNA profiling of hESC cardiac induction suggested that a novel set of miRNAs, many of which were not previously linked to cardiac development and function, contribute to the initiation of cardiac fate switch of pluripotent hESCs [6].

Although RA-induced hESC neuronal derivatives retain an embryonic acetylated globally active chromatin state, NAM induced global histone deacetylation, significant down-regulation of the expression of Brg-1 and HDAC1, and nuclear translocation of the class III NAD-dependent histone deacetylase SIRT1 [6]. This observation suggests that NAM triggers the activation of SIRT1 and NAD-dependent histone deacetylation that lead to globalchromatin silencing yet selective activation of a subset of cardiac-specific genes, and subsequently cardiac fate determination of pluripotent hESCs [6]. Sir2 and its human orthologue SIRT1 are members of the sirtuin family and class III NAD-dependent HDAC [19]. These enzymes catalyze a unique reaction in which NAD and acetylated histone are converted into deacetylated histone, NAM, and a novel metabolite *O*-acetyl ADP-ribose (*O*AADPr) [19]. NAM acts as a noncompetitive product inhibitor of the forward deacetylation reaction of NAD-dependent SIRT1 and is likely regulating SIRT1 activity *in vivo* [19,145,146]. In humans, there are 7 homologues (SIRT1-7) among which SIRT1, 6, 7 are classified as nuclear sirtuins, and SIRT2 as cytoplasmic sirtuin, whereas SIRT3, 4, 5 reside in the mitochondria [147]. SIRT1, 2, 3, 5 are NAD-dependent histone/protein deacetylases, whereas SIRT4, 6 are primarily mono-ADP-ribosyl transferases and SIRT7 exhibits phosphoribosyl-transferase with no deacetylase activity *in vitro* [147]. NAD-dependent SIRT1, which has long been considered as the anti-aging target, is a critical epigenetic regulator previously implicated in cardiovascular and metabolic diseases as well as during embryogenesis [64,147-151]. SIRT1 is expressed at high levels in the heart and the nervous system during embryogenesis, suggesting that it is a critical epigenetic regulator in embryogenesis [64, 148]. The implication of sirtuins as potential pharmacological targets has resulted in a firestorm of work on the seven mammalian sirtuins in less than a decade, however, the important connection between the histone deacetylase activity of SIRT1 and chromatin has been underappreciated. SIRT1 mediates deacetylation of histones, in particular histone H4 K16, and the recruitment of the linker histone H1 [132]. SIRT1 also promotes histone H3 K9 methylation by its direct recruitment of HMT SUV39H1 and by elevating SUV39H1 activity through conformational changes and deacetylation of SUV39H1 in its SET domain, concomitant with heterochromatin formation [132]. SIRT1 plays an essential role in heterochromatin silencing and euchromatic repression in mammalian development through association with the bHLH repressor proteins and histone rearrangement [19,63,132,152]. Of the four lysine residues in the N-terminal tail of H4 (K5, 8,12, 16), K16 is the specific target of SIRT1 and plays a unique role in regulating chromatin structure [8,19,132]. Histone H4 K16 acetylation is important in epigenetic regulation as substantiated by its being the only lysine residue among the N-terminal tails of all histones that is targeted by an exclusive category of HATs as well as HDACs, such as the MYST family of HATs and the class III NAD-dependent HADCs to mediate silencing of chromatin locus during phenotype switch in human development [8,19,56,132]. Further unveiling the neucleoprotein complex regulation in hESC cardiac lineage specific progression towards cardiomyocytes mediated by NAD-dependent histone deacetylase SIRT1 will provide critical understanding to the molecular mechanism underlying human embryonic cardiogenesis, thereby aid the development of more effective and safe stem cell-based therapeutic approaches in the heart field.

## 5. FUTURE PROSPECTIVES

To date, the lack of a suitable human neuronal or cardiomyocyte source with adequate CNS or myocardium regenerative potential has been the major setback for CNS or myocardial tissue engineering and for developing safe and effective cell-based therapies. Recent technology breakthrough enables direct conversion of pluripotent hESCs into a large supply of high purity neuronal cells or heart muscle cells with adequate capacity to regenerate CNS neurons and contractile heart muscles for developing safe and effective stem cell therapies [5-8,25,32-36]. Such hESC neuronal and cardiomyocyte therapy derivatives provide currently the only available human cell sources with adequate capacity to regenerate CNS neurons and contractile heart muscles, vital for CNS and heart repair in the clinical setting. Lineage-specific differentiation direct from the pluripotent state of hESCs by small molecule induction offers much-needed *in vitro* hESC model systems for investigating molecular controls in human embryonic development as well as a large supply of clinical-grade human neuronal and cardiomyocyte therapy derivatives for CNS and myocardial tissue engineering and cell therapies [5-8,25,32-36]. Studies to profile novel hESC models of human embryonic neurogenesis and cardiogenesis using genome-wide approaches have begun to reveal genetic and epigenetic programs in hESC neuronal and cardiac lineage specification [6,8,34]. Such genome-wide high-resolution mapping will generate comprehensive knowledge of developmental regulators and networks underlying hESC neuronal or cardiac specification for systems biology approaches and network models of human embryogenesis. One of the major challenges in developing hESC therapies is to determine the necessary molecular and cellular cues that direct efficient and predicable lineage-specific differentiation of pluripotent hESCs. The normal human developmental pathways that generate cardiomyocytes and most classes of CNS neurons remain poorly understood. As a result, directing hESC differentiation along specific pathways in a systematic manner has proved difficult. Unveiling genetic and epigenetic programs embedded in hESC lineage specification will not only contribute tremendously to our knowledge regarding molecular embryogenesis in human development, but also allow direct control and modulation of the pluripotent fate of hESCs when deriving an unlimited supply of clinically-relevant lineages for regenerative medicine. Embedding lineage-specific genetic and epigenetic developmental programs into the open epigenomic landscape of pluripotent hESCs will offer a new repository of human stem cell therapy derivatives for the future of regenerative medicine. The outcome of such research programs will potentially shift current research to create new scientific paradigms for developmental biology and stem cell research.

## 6. CONCLUSION

The broad potential of pluripotent hESCs is defined by an epigenome constituted of open conformation of chromatin. Recent technology breakthrough enables direct conversion of pluripotent hESCs by small molecule induction into a large supply of lineage-specific neuronal cells or heart muscle cells with adequate capacity to regenerate neurons and contractile heart muscles. Nuclear translocation of NAD-dependent histone deacetylase SIRT1 and global chromatin silencing lead to hESC cardiac fate determination, while silencing of pluripotence-associated hsa-miR-302 family and drastic up-regulation of neuroectodermal Hox miRNA hsa-miR-10 family lead to hESC neural fate determination. Embedding lineage-specific genetic and epigenetic programs into the open epigenomic landscape of pluripotent hESCs offers a new dimension for direct control and modulation of hESC pluripotent fate when deriving clinically-relevant lineages for regenerative therapies.

## ABBREVIATIONS

5mC: 5-methylcytosine
5hmC: 5-droxymethylcytosine
acH3/4: Acetylated Histone H3/4
CHD: Chromodomain Helicase DNA Binding Protein
CNS: Central Nervous System
ChIP/NuIP-chip: Chromatin/Nucleosome-Immunoprecipitation-Coupled DNA Microarray Analysis
ChIP-seq: Chromatin-Immunoprecipitation-Combined Second-Generation High-Throughput Sequencing
Dmnt: DNA Cytosine Methyltransferases
EB: Embryoid Body
Esa1: Essential Sas2-Related Acetyltransferase
ESC: Embryonic Stem Cell
EZH2: Enhancer of Zeste Homlog 2
HAT: Histone Acetyltransferase
H3K4/9/27me: Methylation of K4/9/27 of Histone H3
HBO1: Histone Acetyltransferase bound to ORC
HDAC: Histone Deacetylases
hESC: Human Embryonic Stem Cell
hESC-I hNu: Human Neuron Induced From Human Embryonic Stem Cell
hESC-I hNuP: Human Neuronal Progenitor Induced From Human Embryonic Stem Cell
HMT: Histone Methyltransferases
hNSC: Human Neural Stem Cell
HP1: Heterochromatin Protein 1
ICM: Inner Cell Mass
iPS Cell: Induced Pluripotent Stem Cell
MBD: Methyl-CpG-binding protein
MI: Myocardial Infarction
miRNA: microRNA
MORF: MOZ-Related Factor
MOZ: Monocytic Leukemia Zinc Finger Protein
NAM: Nicotinamide
NAD: Nicotinamide Adenine Dinucleotide
NSC: Neural Stem Cell
NURD: Nucleosomal Remodeling and Deacetylation Complex
OAADPr: O-acetyl ADP-ribose
PcG: Polycomb Group Proteins
RA: Retinoic Acid
Sas: Something About Silencing
Sir2: Silent Information Regulator
Tet: Ten-Eleven Translocation proteins
Tip60: Tat interactive protein 60
trxG: Trithorax Group Proteins
